# The protective effects of the β3 adrenergic receptor agonist BRL37344 against liver steatosis and inflammation in a rat model of high-fat diet-induced nonalcoholic fatty liver disease (NAFLD)

**DOI:** 10.1186/s10020-020-00164-4

**Published:** 2020-06-05

**Authors:** Ziwen Wang, Shanshan Li, Ruifeng Wang, Liansheng Guo, Dan Xu, Tieyuan Zhang, Yifan Xu, Wenlong Wang, Min Wang, Zhongwei Gan, Xiaobing Wang

**Affiliations:** 1 Gastroenterology Department, the First Affiliated Hospital of Harbin Medical University, #23 Postal Street, Harbin, 150001 Heilongjiang China; 2 Gastroenterology Department, the Fourth Affiliated Hospital of Harbin Medical University, #37 Yiyuan Street, Harbin, 150001 Heilongjiang China; 3 Harbin Medical University, #157 Baojian Street, Harbin, 150081 Heilongjiang China

**Keywords:** β3-adrenergic receptor, NAFLD, Liver steatosis, Inflammation

## Abstract

**Background:**

Our objective was to investigate the efficacy of the beta-3 adrenergic receptor (β3-AR) agonist BRL37344 for the prevention of liver steatosis and inflammation associated with nonalcoholic fatty liver disease (NAFLD).

**Methods:**

Four groups were established: a control group (given a standard diet), a high-fat diet (HFD) group, an HFD + β3-AR agonist (β3-AGO) group, and an HFD + β3-AR antagonist (β3-ANT) group. All rats were fed for 12 weeks. The β3-AR agonist BRL37344 and the antagonist L748337 were administered for the last 4 weeks with Alzet micro-osmotic pumps. The rat body weights (g) were measured at the end of the 4th, 8th, and 12th weeks. At the end of the 12th week, the liver weights were measured. Serum alanine aminotransferase (ALT) and aspartate aminotransferase (AST) were analyzed with a Hitachi automatic analyzer. The lipid levels of the triglycerides (TGs), total cholesterol (TC), and low-density lipoprotein cholesterol (LDL-C) and the concentrations of free fatty acids (FFAs) were also measured. An oil red O kit was used to detect lipid droplet accumulation in hepatocytes. Steatosis, ballooning degeneration and inflammation were histopathologically determined. The protein and mRNA expression levels of β3-AR, peroxisome proliferator-activated receptor-alpha (PPAR-α), peroxisome proliferator-activated receptor-gamma (PPAR-γ), mitochondrial carnitine palmitoyltransferase-1 (mCPT-1), and fatty acid translocase (FAT)/CD36 were measured by western blot analysis and RT-qPCR, respectively.

**Results:**

After treatment with the β3-AR agonist BRL37344 for 4 weeks, the levels of ALT, AST, TGs, TC, LDL-C and FFAs were decreased in the NAFLD model group compared with the HFD group. Body and liver weights, liver index values and lipid droplet accumulation were lower in the HFD + β3-AGO group than in the HFD group. Decreased NAFLD activity scores (NASs) also showed that liver steatosis and inflammation were ameliorated after treatment with BRL37344. Moreover, the β3-AR antagonist L748337 reversed these effects. Additionally, the protein and gene expression levels of β3-AR, PPAR-α, and mCPT-1 were increased in the HFD + β3-AGO group, whereas those of PPAR-γ and FAT/CD36 were decreased.

**Conclusion:**

The β3-AR agonist BRL37344 is beneficial for reducing liver fat accumulation and for ameliorating liver steatosis and inflammation in NAFLD. These effects may be associated with PPARs/mCPT-1 and FAT/CD36.

## Background

Nonalcoholic fatty liver disease (NAFLD), a multifactorial clinical pathological syndrome characterized by lipid accumulation in hepatocytes and hepatocyte steatosis, includes fatty liver alone and nonalcoholic steatohepatitis (NASH); NASH can slowly progress into liver fibrosis, cirrhosis and even liver cancer (Berlanga et al. 2014; Perumpail et al. 2015). The latest research (Bhatia et al. 2012) has shown that NAFLD can also increase the risk of mortality associated with cardiovascular disease. With the rapid development of the economy, people are consuming fewer vegetables and eating more meat. Fatty meat and animal visceral food contain a large amount of saturated fat and cholesterol. In addition, with the acceleration of work and life rhythm, people are more likely to choose fried food and increase the intake of sweets, and they are rich in saturated fat. Sugars can be stored in the body in the form of glycogen and converted into fat. Moreover, with the popularity of mobile devices, many people are infatuated with mobile phones, iPads and other electronic devices. The development of artificial intelligence has caused many physical tasks to be replaced by machines. As a result, less exercise leads to increased fat storage, an increased incidence of NAFLD with time, and a decreased age at onset (Satapathy and Sanyal 2015). However, the underlying pathogenesis of NAFLD is still not fully understood.

The main function of the beta adrenergic receptor (β-AR) is to regulate cardiac function through the sympathetic nervous system (Dessy and Balligand 2010; Balligand 2013). However, studies have shown that the expression of beta 1 and beta 2 receptors increases with age and promotes liver lipid accumulation (Ghosh et al. 2012). Beta-3 adrenergic receptor (β3-AR), unlike the traditional beta-1 adrenergic receptor (β1-AR) and beta-2 adrenergic receptor (β2-AR), was first identified in adipocytes and plays roles in lipolysis and pyrogenesis in brown adipose tissue (Langin et al. 1995). Considerable research suggests that sympathetic nervous activation and abnormal signal transduction of β3-AR play significant roles in the onset and development of metabolic syndromes such as obesity, hypertension (Dessy and Balligand 2010), diabetes, and hyperlipidemia (Masuo 2010). NAFLD is a metabolic stress-related liver injury disease closely related to insulin resistance (IR) and genetic susceptibility and is considered a liver manifestation of metabolic syndrome. Moreover, NAFLD is a risk factor for type 2 diabetes, dyslipidemia and hypertension. However, the relationship between the expression of β3-AR and the occurrence and development of NAFLD has not been reported. Studies published in Hepatology (Trebicka et al. 2009) and the British Journal of Pharmacology (Vasina et al. 2012) have revealed that the expression of β3-AR is significantly increased in liver fibrosis patients and carbon tetrachloride (CCl_4_)-induced rat models of liver fibrosis and that portal vein pressure is regulated through the adenylate cyclase (β3-AR/cyclic adenosine monophosphate) pathway. The liver pathological changes caused by CCl_4_ are similar to those occurring in NAFLD, such as steatosis, ballooning degeneration, inflammatory cell infiltration and fibrosis. Therefore, we hypothesized that liver β3-AR is upregulated when the sympathetic nervous system is activated in the context of NAFLD and that application of the β3-AR agonist BRL37344 will reduce lipid accumulation and inflammation in the liver. We speculate that upregulation of β3-AR expression following sympathetic nervous system overactivation is a protective mechanism against NAFLD.

## Methods

### Animals

A total of 32 male Sprague-Dawley rats (weight 200 ± 20 g) were obtained from the Animal Research Center of Harbin Medical University and raised in a specific pathogen-free animal center (12-h daylight cycle, temperature: 18 °C-22°C) with free access to food and water. After acclimation for 1 w, the rats were randomized into four groups: ① a normal (control) group (*n* = 8) fed a standard diet containing 5% fat, 66% carbohydrate and 23% protein with added fiber, vitamins and minerals; ② a high-fat diet (HFD) group (*n* = 8) fed an HFD consisting of 88% standard feed, 10% lard, and 2% cholesterol for 12 weeks (calorific value 4.8 kcal/g, with an energy composition of 52% from carbohydrate, 30% from fat, and 18% from protein); ③ an HFD + β3-AR agonist (HFD + β3-AGO) group (*n* = 8); and ④ an HFD + β3-AR antagonist (HFD + β3-ANT) group (*n* = 8). Alzet micro-osmotic pumps were subcutaneously implanted into the HFD rats of the HFD + β3-AGO and HFD + β3-ANT groups at the end of the 8th week, and the β3-AR agonist BRL37344 (4.5 μg/(kg·h), Sigma-Aldrich B169) and the β3-AR antagonist L748337 (6 μg/(kg·h), Sigma-Aldrich L7045), respectively, were given continuously for 4 weeks. At the end of the 12th week, all rats were anesthetized through intraperitoneal injection of 10% chloral hydrate (0.3 ml/100 g) and euthanized. Femoral artery blood was collected for analysis of biochemical indicators. Liver samples were collected, snap-frozen in liquid nitrogen and then divided into two portions: the samples in the first were fixed with 10% paraformaldehyde and embedded in paraffin for morphological evaluation, and those in the second were frozen in liquid nitrogen for subsequent assays.

### Body and liver weight measurement

The body weights (g) of the rats in each group were recorded before the procedure and at the end of the 4th, 8th, and 12th weeks. The livers were weighed immediately after excision, and the liver weight/final rat body weight × 100% was calculated as the liver index.

### Biochemical analyses

At the end of the 12-week procedure, rat femoral blood was taken to detect liver function. After blood coagulation, the samples were centrifuged for 10 min at 1000×*g* to separate the serum, and the serum was stored at − 20 °C. Serum alanine aminotransferase (ALT) and aspartate aminotransferase (AST) were analyzed with a Hitachi automatic analyzer (Hitachi 737, Tokyo, Japan). The levels of blood lipids, specifically triglycerides (TGs), total cholesterol (TC), and low-density lipoprotein cholesterol (LDL-C), were also measured.

The frozen liver tissues were powdered in liquid nitrogen and homogenized with 10 mmol/L HEPES and 250 mmol/L sucrose (pH 7.4, 9 vol/g wet tissue) by sonication (Rametta et al. 2013). Lipid was extracted from the livers according to the method of Bligh (Bligh and Dyer 1959; Zhang et al. 2013). TC and TGs were analyzed by flame ionization and quantified by comparing the results with the internal parameters and calibration curves of purified TC and TGs.

### Free fatty acid (FFA) levels in plasma and liver tissues

The assay principle in this study is based on FFA, considering that copper ions may combine to form fatty acid copper salt, soluble in chloroform. Its content is in direct proportion to free fatty acids. The content of FFA can be calculated by measuring the content of copper ions with copper reagent by colorimetry.

All procedures were conducted in accordance with the instructions of an FFA concentration measurement kit (Jiancheng, Nanjing, China). Blood samples were centrifuged at 2000×*g* for 5 min to obtain plasma. Frozen liver tissue was homogenized in a saline buffer and centrifuged at 10000×*g* for 5 min. Plasma and tissue FFAs were measured at 440 and 636 nm, respectively, by spectrophotometer colorimetry.

### Histomorphological analysis

#### Hematoxylin and eosin (HE) staining

Fresh liver tissue was prepared into 1.0 × 0.5 × 0.3 cm^3^ samples and fixed in a 10% buffered formaldehyde solution for 24 h. The fixed specimens were embedded in paraffin after dehydration and clearing and were then made into 5-μm-thick sections for HE staining. All the sections were evaluated under a light microscope by one expert pathologist blinded to the treatments, and the NAFLD activity scores (NASs), proposed by Kleiner et al. (2005) were used to assess the liver histological score. Lobular inflammation was scored from 0 to 3 (0: no foci, 1: < 2 foci, 2: 2–4 foci, 3: > 4 foci), steatosis was scored from 0 to 3 (0: < 5%, 1: 5–33%, 2: 34–66%, 3: > 66%), and ballooning degeneration was scored from 0 to 2 (0: none, 1: few, 2: many). Two slices were taken from each sample, and three different views were selected from each slice (200×). Liver fibrosis was not evaluated due to the short modeling period.

### Oil red O staining

An oil red O kit (Jiancheng, Nanjing, China) was used to detect lipid droplet accumulation in hepatocytes. The liver tissue was frozen and sectioned at a thickness of 10 μm with a frozen slicer (Leica CM1850). According to the instructions of the oil red O staining kit, the lipid droplets were dyed red, and the nuclei were stained dark blue. The lipid droplets were quantified using Image-Pro Plus 6.0 software.

### Electron microscopy

Liver samples were pretreated with 2.5% gluteraldehyde immediately after the removal, cut into the ultrathin sections (50–100 nm), prepared according to the method of Huang et al. (2018) and observed by two professionals blinded to the treatments with the electron microscopy (Hitachi, H7650, Japan).

### Western blotting

Total protein was extracted from liver tissues and quantified by the BCA method. Fifty micrograms of protein was loaded onto a 10% SDS-PAGE gel for electrophoresis. The membranes were routinely washed and blocked with 5% nonfat dry milk in PBST (containing 0.05% Tween 20), oscillated at room temperature for 1 h, and incubated overnight at 4 °C with β3-AR (Santa Cruz, sc-515763, 1:500 dilution), peroxisome proliferator-activated receptor-alpha (PPAR-α) (Santa Cruz, sc-398,394, 1:100 dilution), peroxisome proliferator-activated receptor-gamma (PPAR-γ) (Santa Cruz, sc-81,152, 1:500 dilution), mitochondrial carnitine palmitoyltransferase-1 (mCPT-1) (Abcam, Cambridge, ab104662, 1:100 dilution) and CD36 (Santa Cruz, SC-7309, 1:100 dilution) primary antibodies. After rinsing in TBS, the membranes were incubated with secondary antibodies and oscillated at room temperature for 1 h. Glyceraldehyde-3-phosphate dehydrogenase (GAPDH) levels were measured as an internal control with anti-GAPDH (1:1000) antibodies (Zhongshan Golden Bridge Bio, China). A Bio-Rad imaging system and ImageJ software were used to detect the immunoreactive bands and to quantify each sample.

### Real-time RT-PCR

Total RNA was isolated by TRIzol extraction according to the manufacturer’s instructions. Reverse transcription was performed in 20 μl reaction mixtures with SYBR® Green qPCR reagents and a TaKaRa PrimeScript™ RT Reagent Kit with gDNA Eraser (Code No. RR047A) according to the manufacturer’s recommendations. An ABI 7500 Real Time PCR system (Applied Biosystems) was used to perform quantitative RT-PCR. The steady-state mRNA levels of β3-AR, PPAR-ɑ, PPAR-γ, mCPT-1 and fatty acid translocase (FAT)/CD36 were measured. The specific primers used for gene amplification in the present study are listed in Table 1. GAPDH was used as an internal control.

**Table 1:** Primers for real-time RT-PCR

Gene name	Primer Sequence (5′ → 3′)
β3-AR	Forward: GTGTCCTTTGCGCCCATC
	Reverse: GCGCTTAGCTACGACGAACA
PPAR-α	Forward: CGCTGGGTCCTCTGGTTGTC
	Reverse: TTCAGTCTTGGCTCGCCTCT
PPAR-γ	Forward: TTCGCTGATGCACTGCCTAT
	Reverse: GTCAGCTCTTGTGAACGGGA
CD36	Forward: GGAACTGTGGGCTCATTACTGG
	Reverse: GACAACTTCCCTTTTGATTGTCTTCT
mCPT-1	Forward: GGAGAGTGCCAGGAGGTCATAG
	Reverse: TGTCCTTTGTAATGTGCGAGCTG
GAPDH	Forward: CCTCTGACTTCAACAGCGACAC
	Reverse: TGGTCCAGGGGTCTTACTCC

*β3-AR* beta-3 adrenergic receptor, *PPAR-ɑ* peroxisome proliferator-activated receptor-alpha, *PPAR-γ* peroxisome proliferator-activated receptor-gamma, *mCPT-1* mitochondria carnitine palmitoyltransferase-1, *GAPDH* glyceraldehyde-3-phosphate dehydrogenase

### Statistical analysis

All data are expressed as the mean ± standard deviation (SD). The NASs used to evaluate liver pathological changes were assessed using the Mann-Whitney test (SPSS18.0, Inc., Chicago, IL, USA). Group data were compared using one-way ANOVA with Tukey’s post hoc test for multiple comparisons. A *P* value less than 0.05 was considered to indicate statistical significance. GraphPad Prism 6.0 (La Jolla, CA) was used for the statistical analyses.

## Results

### Upregulation of the protein and mRNA expression of β3-AR in the livers of model rats with HFD-induced NAFLD

**Fig. 1:**
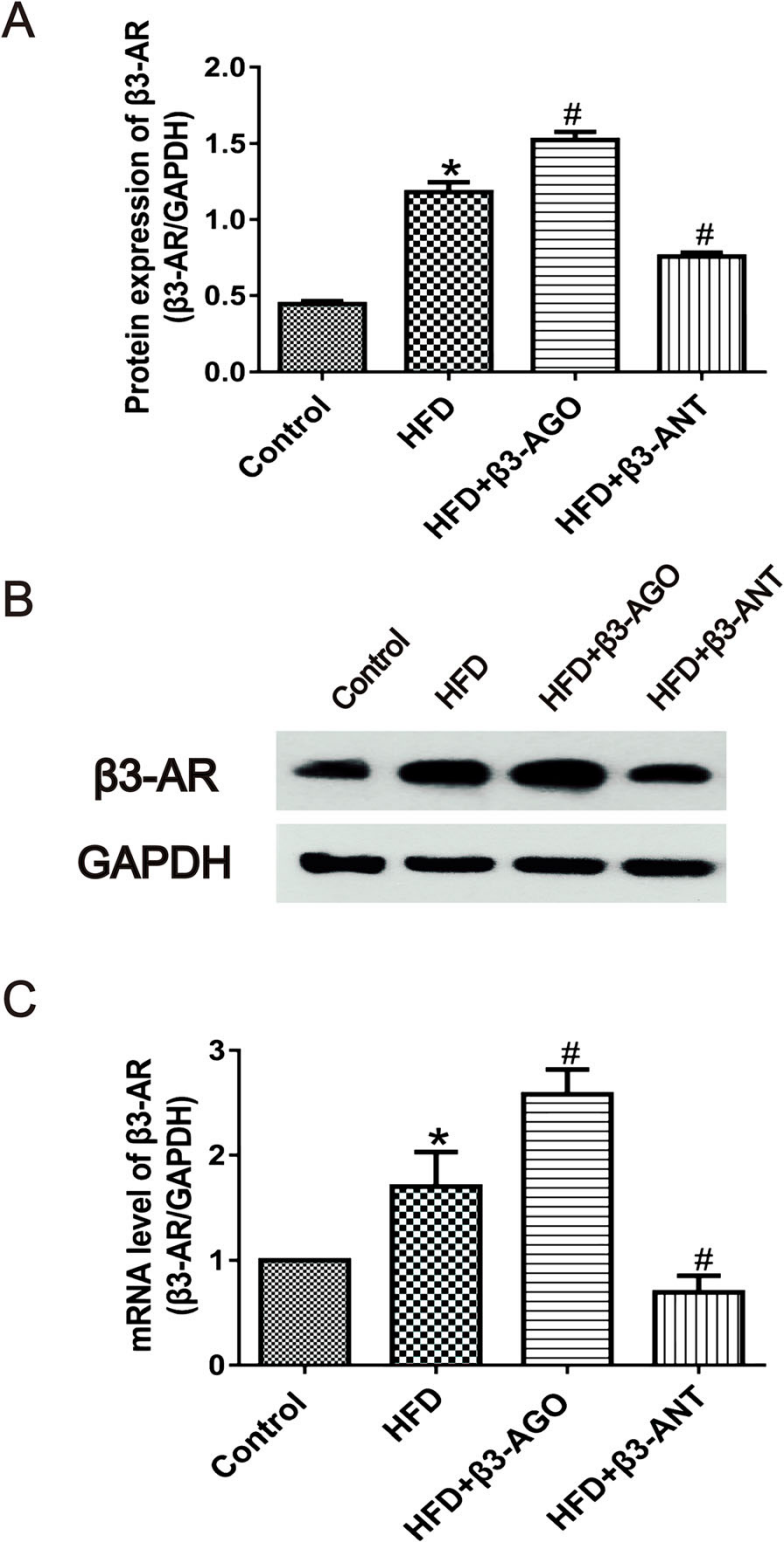
Protein and mRNA expression of β3-AR in the liver in the four groups. **a** Quantitative analysis of β3-AR protein levels relative to GAPDH levels (each group, *n* = 8). **b** Western blot analysis of β3-AR protein levels using specific β3-AR antibodies. GAPDH was used as a loading control. **c** β3-AR mRNA expression in the control, HFD, HFD + β3-AGO and HFD + β3-ANT groups (each group, *n* = 8). β3-AGO refers to the β3-AR agonist BRL37344, and β3-ANT refers to the β3-AR antagonist L748337. *P < 0.05 *vs* the control group, #*P* < 0.05 *vs* the HFD group

As shown in Fig. 1, western blot and real-time PCR analyses demonstrated that the protein and mRNA expression of β3-AR in HFD-fed rat livers was significantly higher than that in control rat livers (*P* < 0.05).

In addition, the protein and mRNA levels of β3-AR were upregulated by BRL37344 and downregulated by L748337 in HFD-fed rat livers (*P* < 0.05).

### β3-AR stimulation decreased the serum ALT, AST, TGs, TC, LDL-C and FFA levels in the livers of model rats with HFD-induced NAFLD

As summarized in Table 2, after 12 weeks, the serum ALT, AST, TGs, TC, LDL-C and FFA levels in rat livers were higher in the HFD group than in the control group (*P* < 0.05). Application of BRL37344 for 4 weeks significantly decreased the serum ALT, AST, TGs, TC, LDL-C and FFA levels in the HFD-fed rats (*P* < 0.05). In contrast, the serum ALT, AST, TGs, TC, LDL-C and FFA levels among rats were markedly higher in the HFD + β3-ANT group (treated with L748337) than in the HFD group (*P* < 0.05).

**Table 2:** Serum biochemical parameters of rats in the control, HFD, HFD + β3-AGO and HFD + β3-ANT groups

	Control(*n* = 8)	HFD (*n* = 8)	HFD + β3-AGO(*n* = 8)	HFD + β3-ANT(*n* = 8)
ALT (U/L)	36.56 ± 2.17	62.84 ± 7.73^*^	46.09 ± 1.75^†^	80.98 ± 5.63^†^
AST (U/L)	145.61 ± 11.9	205.80 ± 3.71^*^	163.14 ± 5.3^†^	232.06 ± 6.9^†^
TG (mmol/L)	0.37 ± 0.05	0.71 ± 0.05^*^	0.49 ± 0.07^†^	0.96 ± 0.10^†^
TC (mmol/L)	1.46 ± 0.06	2.71 ± 0.29^*^	2.34 ± 0.20^†^	3.17 ± 0.12^†^
LDL-C (mmol/L)	0.37 ± 0.05	0.65 ± 0.078^*^	0.50 ± 0.03^†^	0.80 ± 0.08^†^
FFA (mmol/L)	0.63 ± 0.05	0.88 ± 0.068^*^	0.75 ± 0.04^†^	1.03 ± 0.07^†^

*ALT* alanine aminotransferase, *AST* aspartate aminotransferase, *TG* triglyceride, *TC* total cholesterol, *LDL-C* low-density lipoprotein cholesterol, *FFA* free fatty acid. ^*^P < 0.05 vs the control group. ^†^*P* < 0.05 vs the HFD group

### β3-AR stimulation protected against liver steatosis and inflammation in the HFD-induced NAFLD model

**Fig. 2:**
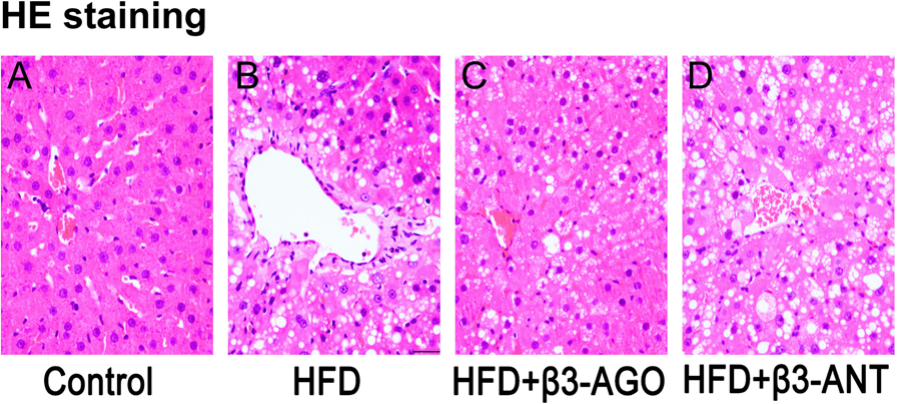
Histological changes in the liver in the control, HFD, HFD + β3-AGO and HFD + β3-ANT groups (each group, *n* = 8). **a** The control group showed normal liver histological features, and the hepatocytes were arranged in an orderly manner. **b** In the HFD group, prominent hepatic steatosis, inflammation and ballooning degeneration were observed. **c** In the HFD + β3-AGO group, significantly improved structural changes with limited hepatic steatosis, inflammation and ballooning degeneration were observed. **d** The image for the HFD + β3-ANT group shows several scattered balloon cells that are much larger than the surrounding steatotic hepatocytes, in sharp contrast to the normal hepatocytes in the image in (**a**). The magnification is 200 ×

The occurrence of steatohepatitis in the control and test groups was investigated by liver histological examination with HE staining (Fig. 2). Treatment of HFD rats with BRL37344 ameliorated liver steatosis, inflammation and ballooning degeneration (Fig. 2c) caused by the HFD (Fig. 2b). However, L748337 aggravated hepatic pathological damage (Fig. 2d).

Table 3 shows that there was no obvious evidence of steatosis or inflammation in the control group. The NASs of the HFD group were significantly higher than those of the control group for liver steatosis, inflammation and hepatocyte ballooning degeneration (Table 3, *P* < 0.05). After 4 weeks of BRL37344 administration, the NASs for liver steatosis, inflammation and hepatocyte ballooning degeneration in the HFD + β3-AGO group were significantly lower than those in HFD group (Table 3, *P* < 0.05). In addition, in the HFD + β3-ANT group, inhibition of β3-AR expression with L748337 increased the NASs for liver steatosis, inflammation and hepatocyte ballooning degeneration and aggravated liver pathological damage (Table 3, *P* < 0.05). These results indicate that β3-AR upregulation protects against liver steatosis and inflammation in this HFD-induced rat NAFLD model.

**Table 3:** Histopathological findings in liver tissue

	Control (*n* = 8)	HFD (*n* = 8)	HFD + β3-AGO (*n* = 8)	HFD + β3-ANT (*n* = 8)
Steatosis				
Grade 0	8	0	1	0
Grade 1	35	7	22	1
Grade 2	5	23	19	17
Grade 3	0	18	6	30
Ballooning				
Grade 0	48	7	19	0
Grade 1	0	23	29	18
Grade 2	0	18	0	30
Inflammation				
Grade 0	34	0	12	0
Grade 1	14	6	24	0
Grade 2	0	31	12	18
Grade 3	0	11	0	30

Two sections of each liver specimen were observed and three views of each section were analysed. Therefore, each group had 48 views (8 rats × 2 sections × 3 views) in total. We calculated the view number distributed in grading NAFLD activity scores (NASs) according to the following categories: steatosis (0–3), lobular inflammation (0–3) and ballooning degeneration (0–2). For steatosis, lobular inflammation and ballooning degeneration, *P* < 0.05, HFD vs control; *P* < 0.05, HFD vs HFD + β3-AGO; *P* < 0.05, HFD vs HFD + β3-ANT

### β3-AR stimulation improved dyslipidemia and liver lipid accumulation in HFD-fed rats

**Fig. 3:**
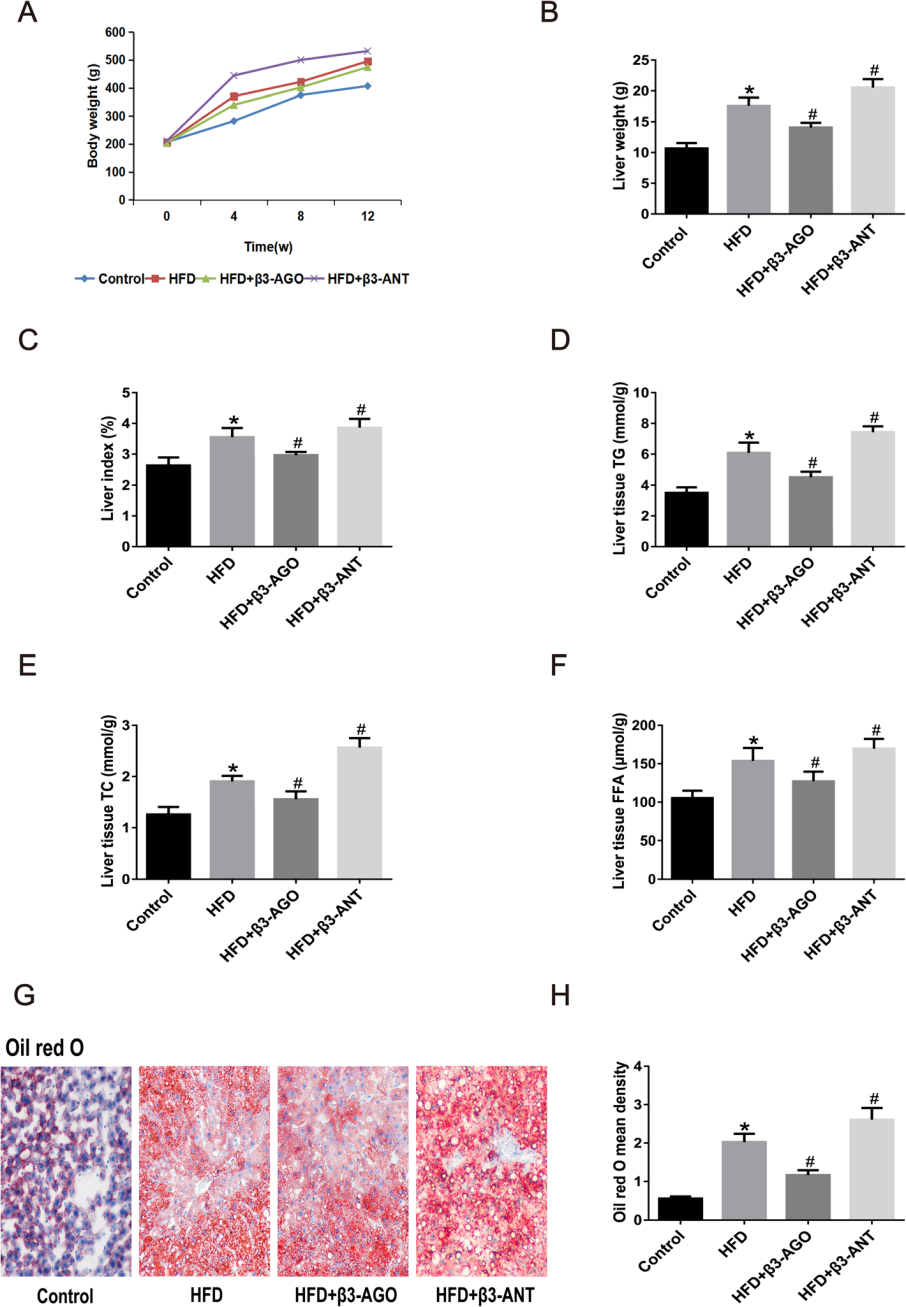
The β3-AR agonist BRL37344 ameliorated HFD-fed rat liver steatosis, exerting a protective effect. **a** Rat body weight at 0, 4, 8, and 12 weeks. **b** Liver weight at the 12th week in the control, HFD, HFD + β3-AGO and HFD + β3-ANT groups. **c** Liver index values at the 12th week in the control, HFD, HFD + β3-AGO and HFD + β3-ANT groups. **d** Liver homogenate TG levels (mmol/g). **e** Liver homogenate TC levels (mmol/g). **f** Liver homogenate FFA levels (μmol/g). **g** Oil red O was used to visualize lipid droplets in the hepatocytes. The magnification is 200×. Lipid droplets are stained light red, and nuclei are stained blue. **h** Quantitative analysis of lipid droplets in hepatocytes. TG: triglyceride. TC: total cholesterol. FFA: free fatty acid. *P < 0.05 vs the control group, #*P* < 0.05 vs the HFD group; *n* = 8 per group

As shown in Fig. 3a, the body weight curve for the HFD group was higher than that for the control group, except at 0 w. By the end of the 12 w procedure, BRL37344 had decreased rat body weight, liver weight and liver index values, whereas L748337 had increased these values (Fig. 3a-c, *P* < 0.05).

In addition, the liver tissue TG, TC and FFA levels were significantly higher in the HFD group than in the control group, but β3-AR treatment reduced the levels of liver TGs, TC and FFAs. Inhibition of β3-AR produced the opposite effect in HFD-fed rats (Fig. 3d-f, *P* < 0.05).

Oil red O staining (Fig. 3g) and quantitative analysis (Fig. 3h) showed that the rats in the HFD group had severe liver steatosis; this steatosis was markedly alleviated in the HFD + β3-AGO group and aggravated in the HFD + β3-ANT group.

These results indicate that β3-AR inhibition significantly increases body weight and disorder of lipid metabolism in HFD-fed rats; in contrast, β3-AR stimulation ameliorates these effects in HFD-fed rats (Fig. 3).

### β3-AR stimulated key FFA β-oxidation-related proteins and genes, including PPAR-ɑ, PPAR-γ, mCPT-1 and CD36, in HFD-fed rat livers

**Fig. 4:**
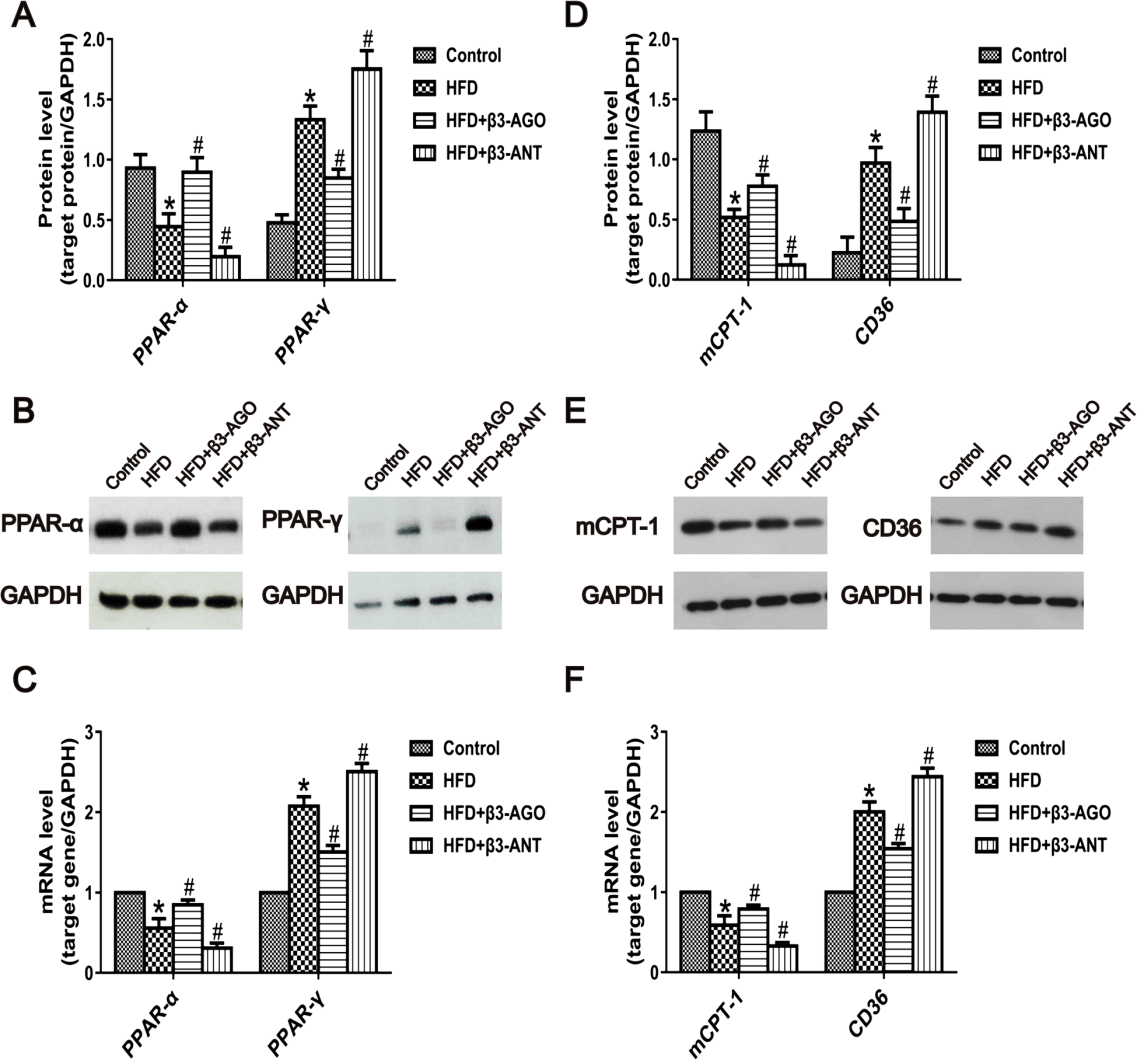
The β3-AR agonist BRL37344 modulated key proteins and genes for FFA β-oxidation. **a** Quantitative analysis of the protein levels of PPAR-α and PPAR-γ in the livers of rats in the control, HFD, HFD + β3-AGO and HFD + β3-ANT groups (each group, *n* = 8). **b** Representative western blot bands of the PPAR-α and PPAR-γ proteins in the livers of rats in the control, HFD, HFD + β3-AGO and HFD + β3-ANT groups. **c** Quantitative analysis of the expression of the PPAR-α and PPAR-γ genes in the control, HFD, HFD + β3-AGO and HFD + β3-ANT groups (each group, *n* = 8). **d** Quantitative analysis of the protein levels of mCPT-1 and CD36 in the livers of rats in the control, HFD, HFD + β3-AGO and HFD + β3-ANT groups (each group, *n* = 8). **e** Representative western blot bands of the mCPT-1 and CD36 proteins in the livers of rats in the control, HFD, HFD + β3-AGO and HFD + β3-ANT groups. **f** Quantitative analysis of the expression of the mCPT-1 and CD36 genes in the control, HFD, HFD + β3-AGO and HFD + β3-ANT groups (each group, *n* = 8). *P < 0.05 vs the control group, #*P* < 0.05 vs the HFD group

Figure 4 shows that the protein and gene expression of PPAR-ɑ, which contributes to FFA β-oxidation, was decreased in the HFD group compared to the control group. Furthermore, BRL37344 upregulated PPAR-ɑ and downregulated PPAR-γ in HFD-fed rat livers, whereas L748337 downregulated PPAR-ɑ and upregulated PPAR-γ (Fig. 4a and c).

mCPT-1 is the key protein regulating the delivery of FFAs into the mitochondria for β-oxidation. Lower protein and mRNA levels of mCPT-1 were observed in the HFD group than in the control group, but activation of β3-AR with BRL37344 increased the mCPT-1 levels. In contrast, inhibition of β3-AR decreased the mCPT-1 levels in HFD-fed rat livers (Fig. 4d and f).

FAT/CD36 is one of the best characterized fatty acid (FA) transporters. The FAT/CD36 level was increased in the HFD group compared to the control group. β3-AR activation inhibited the protein and gene expression of FAT/CD36 in the HFD + β3-AGO group, whereas β3-AR inhibition increased the FAT/CD36 levels in the HFD + β3-ANT group (Fig. 4d and f).

## Discussion

The HFD rat model is prone to liver steatosis and can replicate most of the metabolic characteristics of human NAFLD; thus, this model can be used to investigate NAFLD and related metabolic syndromes (Xu et al. 2010). In the present study, after 12 weeks of HFD feeding (88% standard feed, 10% lard, and 2% cholesterol, calorific value 4.8 kcal/g, with an energy composition of 52% from carbohydrate, 30% from fat, and 18% from protein), body weight, liver weight and liver index values were significantly higher in the HFD group than in the control group. There are some studies using the same or similar diet composition and same duration that have successfully induced steatosis, inflammation, and no fibrosis (Fan et al. 2005a, 2005b; Xu et al. 2010). In addition, serum transaminase and serum and liver lipid levels were abnormal, and typical NAFLD histological features such as steatosis, inflammation and ballooning appeared. These changes indicate that the rat NAFLD model was successfully constructed and applicable for this study.

The latest research has proposed a “multiple parallel hits” hypothesis for the main pathogenetic mechanism of NAFLD. Lipid accumulation in hepatocytes, acting as the “first hit”, makes the liver sensitive to further damage, which is manifested in parallel hits of various combinations of processes such as inflammation, oxidative stress, mitochondrial dysfunction, adipokine secretion, intestinal microflora disruption, and adipose tissue dysfunction (Tilg and Moschen 2010; Duseja and Chawla 2014; Vespasiani-Gentilucci et al. 2015; Carotti et al. 2015). Therefore, it is proposed that FAs and their metabolites may be the true lipotoxic substances that cause the progression of NAFLD. This study preliminarily reveals that activation of β3-AR can alleviate lipid accumulation in hepatocytes in the context of NAFLD and that the specific mechanism is related to key regulatory proteins of FA metabolism. Therefore, it is proposed that activation of β3-AR can interfere with the initiation mechanism of NAFLD. Furthermore, activation of β3-AR was also found to inhibit liver inflammation in NAFLD, which suggests β3-AR as a new therapeutic target for NAFLD.

### β3-AR expression was increased in the livers of NAFLD model rats, playing a protective role

BRL37344 has an approximately 20-fold and 100-fold higher selectivity for β3-AR versus β2-AR and β1-AR, respectively (Hoffmann et al. 2004). The affinity of L748337 to β3-AR was also higher than that of β1- or β2-ARs (Candelore et al. 1999). β3-AR has pharmacodynamic characteristics unique from those of β1-AR and β2-AR. During long-term sympathetic nervous system activation, the responses of β1-AR and β2-AR to β-AR agonists decline or even disappear, while β3-AR retains its activity (Langin et al. 1995). Therefore, we overlooked the non-specific effects of BRL37344 and L748337 in this study. β3-AR upregulation in diseases and the resistance of β3-AR to desensitization make it an attractive target for disease treatments. With regard to metabolic diseases, Karimi Galougahi et al. (2016) found that stimulation of β3-AR can ameliorate endothelial dysfunction associated with hyperlipidemia, alleviate vascular dysfunction in diabetic patients, and help prevent cardiovascular complications of diabetes by regulating the redox balance of NO. Accordingly, activation of β3-AR aids in the prevention of hypertension, obesity, diabetes and hyperlipidemia by ameliorating vasodilation, exerting antioxidative effects, and attenuating IR and lipid accumulation (Dessy and Balligand 2010; Xiao et al. 2015). Notably, the incidence of NAFLD is often closely related to obesity, insulin resistance, type 2 diabetes, etc. (Scorletti and Byrne 2016). The results of this study revealed that the protein and gene expression of β3-AR was significantly increased in NAFLD models induced by HFD feeding (Fig. 1), consistent with the increased expression of β3-AR in rats with CCl_4_-induced cirrhosis and in patients with cirrhosis (Trebicka et al. 2009; Vasina et al. 2012). CCl_4_-induced liver injury is mainly characterized by severe hepatocyte steatosis, and the main pathological feature of NAFLD in the liver is also steatosis. Thus, we can speculate that the expression of β3-AR is increased in steatotic livers. Upon activation of β3-AR with BRL37344, the concentrations of ALT, AST, TGs, TC, and LDL-C in plasma and of TGs and TC in liver tissues were significantly decreased in the NAFLD models (Fig. 3 and Table 2). Nevertheless, application of L748337, a β3-AR antagonist, significantly increased ALT, AST, TG, TC and LDL-C levels in plasma and TG and TC levels in liver tissue. In addition, β3-AR activation with BRL37344 decreased body weight, liver weight and liver index values, while treatment with L748337, a β3-AR antagonist, increased body weight, liver weight and liver index values (Fig. 3a-c, *P* < 0.05). The above results indicate that activation of β3-AR can ameliorate gross morphological changes in the liver associated with NAFLD, improve liver function, regulate blood lipid and liver lipid levels, and protect against NAFLD.

### β3-AR stimulation ameliorated steatosis and pathological damage in the livers of NAFLD model rats

In this study, compared with rats in the control group, the rats in the HFD group had significant liver steatosis (Fig. 2, HE staining) and increased lipid droplet accumulation (Fig. 3g and h, *P* < 0.05). In the HFD + β3-AGO group, after application of BRL37344 for 4 weeks, the NASs for liver steatosis and ballooning were significantly lower than those in the NAFLD group (Table 3, *P* < 0.05), and the results of oil red O staining showed that liver lipid droplet accumulation was also significantly decreased (Fig. 3g and h, *P* < 0.05). In contrast, in the HFD + β3-ANT group, liver steatosis and ballooning were significantly aggravated (Table 3, *P* < 0.05), and lipid droplet accumulation was significantly increased (Fig. 3g and h, *P* < 0.05) after application of L748337 4 weeks. Xiao et al. (2015) showed that treatment of C57BL/6 J mice with the β3-AR agonist CL316243 (25 μg/day) for 4 weeks increases energy expenditure and ameliorates glucose tolerance, thus exerting antiobesity effects, through a mechanism related to activation of brown adipose tissue. The results of this study indicate that the upregulation of β3-AR after BRL37344 application plays a protective role, ameliorating pathological damage such as liver steatosis, in the rat model of HFD-induced NAFLD, similar to the results of Cuiying Xiao et al. A recent study (Decara et al. 2018) revealed that treatment with a combination of the β3-AR agonist CL316243 and liraglutide (a human glucagon-like peptide-1 [GLP-1] analog) for one week decreased liver lipid content and serum transaminase and lipid levels in rats, similar to the results of our study. This study used a simple β3-AR agonist for four weeks, and the findings fully confirmed that β3-AR agonism remains effective over a long period of time. The above results suggest that upregulation of β3-AR expression is a protective mechanism against NAFLD and may therefore be a new therapeutic target for NAFLD.

### β3-AR stimulation regulated the expression of peroxisome proliferator-activated receptors (PPARs)/mCPT-1 and FAT/CD36, ameliorated liver lipid accumulation, and helped alleviate liver steatosis in NAFLD (Fig. 5)

Accumulation of TGs in the cytoplasm of hepatocytes stems from imbalances between lipid production (which occurs through FA uptake and lipid regeneration) and depletion (which occurs through oxidation and transduction of mitochondrial FA, a component of very low-density lipoprotein particles) along with a variety of pathophysiological mechanisms. Recent studies have suggested that FAs and their metabolites are the harmful factors during the development of NAFLD (Berlanga et al. 2014). The main pathophysiological mechanism of NAFLD involves increases in FA accumulation and decreases in mitochondrial FA oxidation that lead to hepatocyte lipid metabolism dysfunction and lipid accumulation and to hepatocyte injury resulting from activation of tumor necrosis factor-ɑ and stimulation of reactive oxygen species (ROS) production (Berlanga et al. 2014). A previous study (Liu et al. 2013) has shown that in a rabbit model of atrial fibrillation, β3-AR activation can produce levels of FAs and TGs that exceed the oxidative capacity of FA oxidases, leading to lipid accumulation in cardiomyocytes. In contrast, in this study, the concentrations of FFA in plasma and liver tissue were significantly decreased in the NAFLD models after activation of β3-AR by BRL37344 for 4 weeks (Fig. 3f and Table 2). Application of L748337 significantly increased FFA concentrations in plasma and liver tissue (Fig. 3f and Table 2), resulting in FA accumulation in the liver and aggravation of liver injury. These differences in findings may be related to the operation of different pathways downstream of β3-AR activation in diverse diseases and organs.

**Fig. 5:**
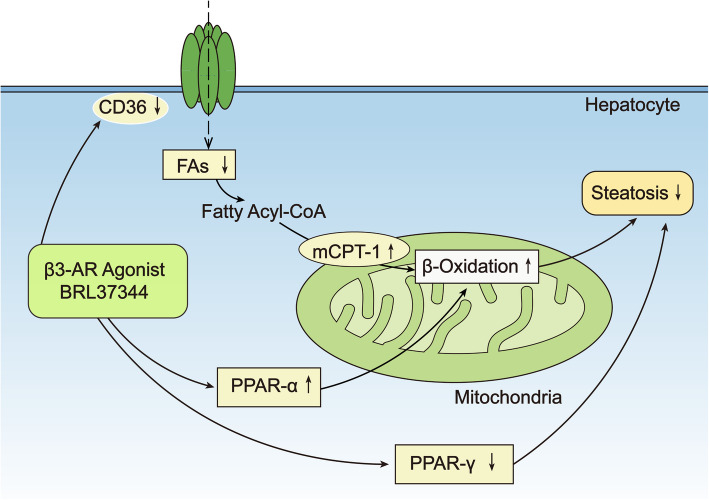
β3-AR stimulation plays a role in alleviating liver steatosis associated with NAFLD. Treatment with the β3-AR agonist BRL37344 for 4 weeks decreased CD36 in NAFLD rats, thus decreasing FAs entry into hepatocytes. On the other hand, increases in mCPT-1 led to increased FAs entry into mitochondria. These two processes together caused a decrease in FA levels in hepatocytes. In addition, the increased FA levels in mitochondria and the increased PPAR-ɑ levels were beneficial for mitochondrial β-oxidation. BRL37344 also decreased PPAR-γ expression. All of the above factors contributed to the relief of liver steatosis in NAFLD rats. β3-AR: beta-3 adrenergic receptor; FAs: fatty acids; mCPT-1: mitochondrial carnitine palmitoyltransferase-1; PPAR-ɑ: peroxisome proliferator-activated receptor-alpha; PPAR-γ: peroxisome proliferator-activated receptor-gamma

The PPAR family comprises three isoforms of ligand-activated nuclear hormone receptors, PPAR-ɑ, PPAR-δ and PPAR-γ (Messmer et al. 2015), that are distributed in various tissues. PPAR-ɑ is widely expressed in the liver and is a major regulator of lipid metabolism. PPAR-ɑ regulates the expression of various genes responsible for lipid metabolism that are also responsible for FA uptake, FA oxidation, cell generation, TG processing and lipid droplet formation and decomposition (Kersten 2014). Previous research (Liu et al. 2013) has shown that stimulating β3-AR in the atria of rats with atrial fibrillation can reduce the FA content of myocardial tissue; decreases in PPAR-ɑ/PPAR-γ coactivator-1 alpha (PGC-1α) levels have also been found, suggesting that PPAR-ɑ/PGC-1ɑ is involved in β3-AR-mediated metabolic remodeling associated with atrial fibrillation. The results of our study showed that the protein and gene expression of PPAR-ɑ related to β-oxidation of FFAs was decreased in the HFD group compared with the control group; however, in HFD-fed rat livers, BRL37344 upregulated PPAR-ɑ, whereas inhibition of β3-AR exerted the opposite effect (Fig. 4a and c). This indicates a possible mechanism in which β3-AR stimulation accelerates FFA oxidation and alleviates lipid accumulation in NAFLD by upregulating the level of PPAR-ɑ. PPAR-γ is the major transcriptional regulator of adipogenesis and is generally believed to have functions opposite those of PPAR-ɑ. Activation of PPAR-γ primarily promotes lipid storage (Liss and Finck 2017). In NAFLD livers, increased expression of PPAR-γ in adipocytes activates lipogenic genes and may contribute to the development of steatosis. The results of this study revealed that the expression of PPAR-γ was increased in the livers of rats with NAFLD. However, BRL37344 application inhibited the expression of PPAR-γ and ameliorated liver steatosis. Nevertheless, it has been reported in some studies that PPAR-γ overexpression has a protective effect against hepatocyte steatosis that may be related to decreased FFA deposition in liver along with increased insulin sensitivity of adipose tissue (Berlanga et al. 2014); these findings are inconsistent with the results in the present study.

mCPT-1 is a regulatory enzyme in mitochondria that facilitates the transfer of FAs from the cytoplasm to the mitochondria for β-oxidation. Inhibition of mCPT-1 has been suggested to prevent IR caused by HFD consumption by reducing some harmful intermediates produced by incomplete FA oxidation and by increasing energy supply for glucose oxidation (Huang et al. 2013). This study demonstrated that mCPT-1 expression was significantly decreased in NAFLD rats and that β3-AR agonist treatment could upregulate mCPT-1 expression, playing a role in reducing NAFLD liver steatosis. Conversely, β3-AR antagonist treatment downregulated mCPT-1 expression (Fig. 4d and f). FAs enter cells by passive diffusion through a protein-mediated mechanism. This process involves a series of FA transporters, of which FAT/CD36 is the most representative (Berlanga et al. 2014). FAT/CD36 is expressed in cells including macrophages, adipocytes, myocytes, intestinal cells and hepatocytes. This transmembrane protein plays an important role in promoting FA uptake and absorption. The intracellular transport and esterification of FFAs, as well as the role of TGs in heart and skeletal muscle cells, are largely dependent on FAT/CD36 translocation from the intercellular milieu to the plasma membrane. Insulin can induce FAT/CD36 translocation and enhance FFA uptake. In NAFLD patients with morbid obesity, Greco et al. (2008) revealed that liver FAT/CD36 mRNA levels are positively correlated with liver lipid content, and another study (Miquilena-Colina et al. 2011) has demonstrated that upregulation of liver FAT/CD36 is significantly correlated with IR, hyperinsulinemia, and increased liver steatosis in the context of NASH. The results of Liu et al. (2013) showed that in a rabbit model of atrial fibrillation, application of a β3-AR agonist for 1 week could inhibit the expression of CD36 and help restore cardiac energy metabolism. The results of this study indicate that β3-AR stimulation can inhibit the expression of CD36 in the livers of NAFLD rats (Fig. 4d and f), which may be one of the mechanisms by which β3-AR helps ameliorate liver steatosis.

### Increased expression of β3-AR ameliorated inflammation in the livers of NAFLD model rats

Inflammatory cell infiltration and upregulation of inflammatory mediators are secondary hits in the pathogenesis of NAFLD. It is generally believed that inflammatory factors cause hepatocyte damage by inducing ROS production and by releasing proteases and inflammatory mediators. New evidence suggests that inflammation plays a key role in promoting the progression of steatohepatitis to liver cirrhosis and liver cancer (Gao and Tsukamoto 2016). This study showed that after administration of BRL37344 to the HFD + β3-AGO group for 4 weeks, the NASs (Grades 2 and 3), which reflects liver inflammation, was significantly lower in the HFD + β3-AGO group than in the HFD group (Fig. 2b-c and Table 3, *P* < 0.05). In addition, in the HFD + β3-ANT group, L748337 inhibited the expression of β3-AR, increased liver inflammation, and aggravated liver pathological damage (Fig. 2d and Table 3, *P* < 0.05). Moreover, many studies on PPAR-ɑ activation in the liver have shown that PPAR-ɑ activation, in addition to regulating metabolic processes, can also reduce liver inflammation caused by the expression of cytokines and other compounds by inhibiting proinflammatory genes (Gervois et al. 2001; Gervois et al. 2004). Moreover, PPAR-γ expression has been shown to have anti-inflammatory and antifibrotic effects in stellate cells, macrophages and epithelial cells (Berlanga et al. 2014). In the present study, β3-AR stimulation activated PPAR-ɑ and inhibited PPAR-γ to exert an antifibrotic effect, perhaps because PPAR-γ expression always shows a trend opposite to that of PPAR-α in the context of excessive fat intake (Fraulob et al. 2012; Nakamura and Terauchi 2013). In addition, among the three subtypes of PPARs, PPAR-ɑ is the most highly expressed subtype in the liver (Liss and Finck 2017). We believe that PPAR-ɑ may play a dominant role during this process.

### β3-AR activation reversed the mitochondrial ultrastructural damage in the livers of NAFLD model rats

As the power of cells, mitochondria play an important role in lipid metabolism. Mitochondrial dysfunction of liver is considered to be the core of the pathogenesis of NAFLD (Khoo et al. 2019). It has been found that HFD can induce liver mitochondrial dysfunction, such as oxidative stress, cytochrome C release, adenosine triphosphate (ATP) metabolism disorder, respiratory reduction, liver mitochondrial ultrastructural abnormality, fatty acid oxidation reduction, inhibit mitosis, and promote the expression of mitochondrial apoptosis related protein (Zhou et al. 2019).

**Fig. 6:**
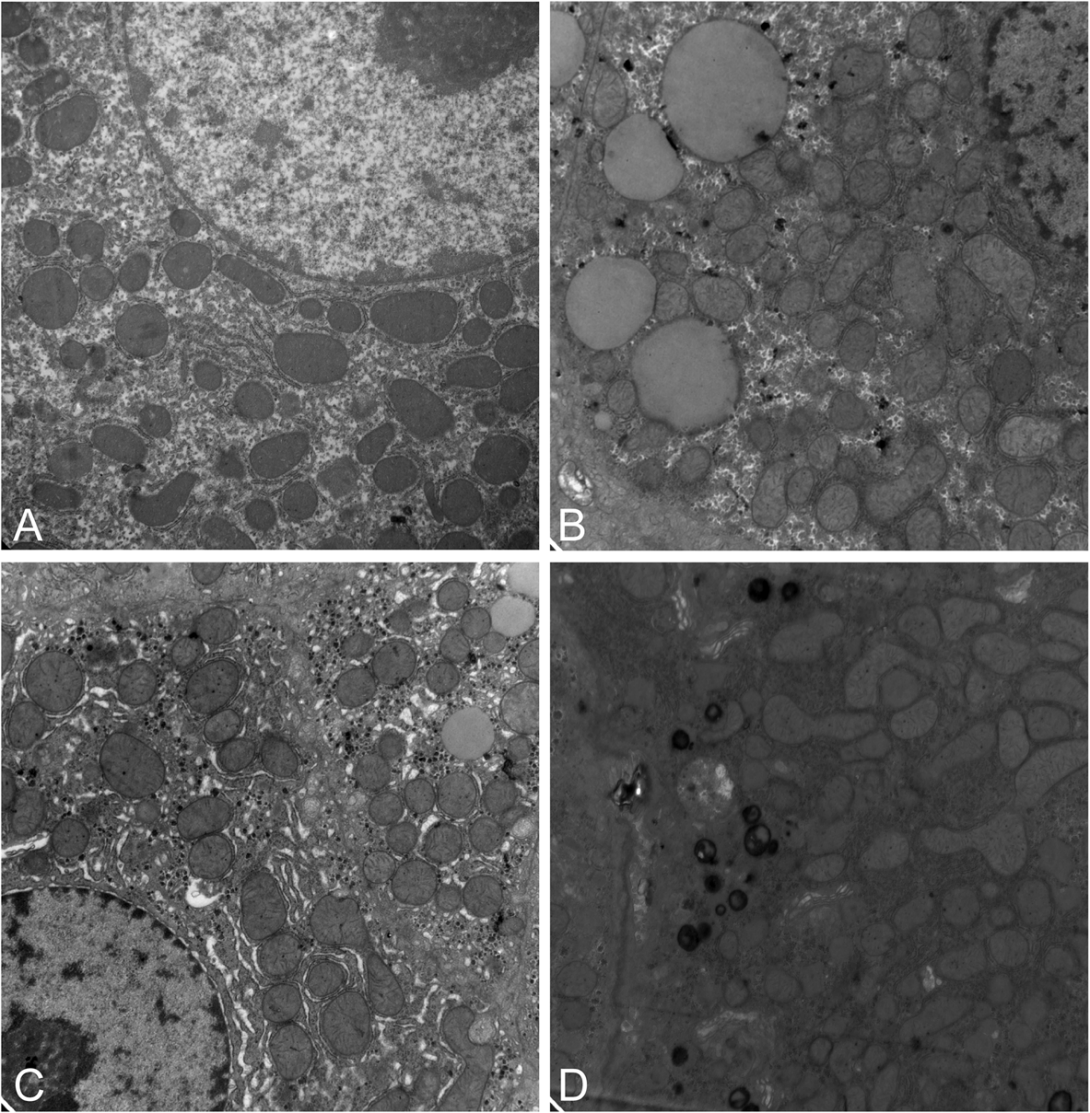
Typical mitochondria changes of liver under transmission electron microscopy. **a** The size of mitochondria was uniformly sized in the control group. **b** Mitochondrial matrix was swollen in the HFD group. **c** Compared with the HFD group, mitochondrial ultrastructural damage was reversed by BRL37344 in the HFD + β3-AGO group. **d** The HFD + β3-ANT group showed more severe mitochondrial swelling and increased lysosomes than that of the HFD group. The magnification was 15,000× in each image (each group, *n* = 8)

Mitochondrial dysfunction impairs lipid metabolism, leads to excessive production of ROS, further inhibits antioxidant capacity, and causes mitochondrial damage (Begriche et al. 2006). In this study, electron microscopy showed that in HFD induced NAFLD model rats, the mitochondrial matrix was swollen (Fig. 6b), while L748337, an inhibitor of β3-AR, aggravated the mitochondrial swelling and increased lysosomes (Fig. 6d). In contrast, BRL37344, an agonist of β3-AR, reversed the mitochondrial ultrastructural damage (Fig. 6c). These findings may preliminarily indicate that the β3-AR activation could alleviate the progression of NAFLD by protecting mitochondria. Therefore, in the next step, we will further observe the activity of the mitochondrial respiratory chain, measure the synthesis of ATP, and evaluate the activity of liver mitochondrial complex I, IV and V to further verify whether the mitochondrial respiratory function is improved due to the activation of β3-AR.

A limitation of this study is related to a previous finding that HFD rats typically show the beginning signs of hepatic perisinusoidal fibrosis at 16 w and develop obvious fibrosis at 24 w (Xu et al. 2010); in this study, the modeling period was 12 w, and no obvious fibrosis was observed. Thus, the effects of β3-AR on liver fibrosis associated with NAFLD could not be evaluated.

## Conclusions

In conclusion, this study demonstrated that the expression of β3-AR is upregulated in NAFLD using an animal model of NAFLD. The experiments involving stimulation and inhibition of the expression of β3-AR have shown, for the first time, that long-term stimulation of β3-AR for 1 month can ameliorate liver lipid accumulation, liver steatosis and inflammation associated with NAFLD. These effects are probably associated with regulation of PPARs/mCPT-1 and FAT/CD36 expression. The findings indicate that upregulation of β3-AR expression is a protective mechanism against NAFLD, suggesting that β3-AR may be a new therapeutic target for NAFLD. In addition, hepatocyte steatosis, inflammation and oxidative stress are interrelated processes. Therefore, it is speculated that β3-AR activation is a complex event that protects against NAFLD. The relationship among β3-AR, liver inflammation and oxidative stress in the context of NAFLD will be the focus of further study.

## Data Availability

All relevant data supporting the conclusions of this article are included within the manuscript.

## Abbreviations

ALT: Alanine aminotransferase
AST: Aspartate aminotransferase
ATP: Adenosine triphosphate
CCl_4_: Carbon tetrachloride
FAs: Fatty acids
FAT/CD36: Fatty acid translocase/CD36
FFA: Free fatty acid
FFAs: Free fatty acids
GAPDH: Glyceraldehyde-3-phosphate dehydrogenase
GLP-1: Glucagon-like peptide-1
HE: Hematoxylin and eosin
HFD: High-fat diet
HFD + β3-AGO: HFD + β3-AR agonist
HFD + β3-ANT: HFD + β3-AR antagonist
IR: Insulin resistance
LDL-C: Low density lipoprotein cholesterol
mCPT-1: Mitochondria carnitine palmitoyltransferase-1
NAFLD: Nonalcoholic fatty liver disease
NASs: NAFLD activity scores
NASH: Nonalcoholic steatohepatitis
PGC-1ɑ: Peroxisome proliferator-activated receptor γ coactivator-1 alpha
PPARs: Peroxisome proliferator-activated receptors
PPAR-ɑ: Peroxisome proliferators-activated receptor-alpha
PPAR-γ: Peroxisome proliferators-activated receptor-Gamma
ROS: Reactive oxygen species
TC: Total cholesterol
TG: Triglyceride
β-AR: Beta adrenergic receptor
β1-AR: Beta-1 adrenergic receptor
β2-AR: Beta-2 adrenergic receptor
β3-AR: Beta-3 adrenergic receptor
